# Diversity and Complexity in Chromatin Recognition by TFII-I Transcription Factors in Pluripotent Embryonic Stem Cells and Embryonic Tissues

**DOI:** 10.1371/journal.pone.0044443

**Published:** 2012-09-10

**Authors:** Aleksandr V. Makeyev, Badam Enkhmandakh, Seung-Hyun Hong, Pujan Joshi, Dong-Guk Shin, Dashzeveg Bayarsaihan

**Affiliations:** 1 Department of Reconstructive Sciences, Center for Regenerative Medicine and Skeletal Development, School of Dentistry, University of Connecticut Health Center, Farmington, Connecticut, United States of America; 2 Computer Science and Engineering, School of Engineering, University of Connecticut, Storrs, Connecticut, United States of America; George Mason University, United States of America

## Abstract

*GTF2I* and *GTF2IRD1* encode a family of closely related transcription factors TFII-I and BEN critical in embryonic development. Both genes are deleted in Williams-Beuren syndrome, a complex genetic disorder associated with neurocognitive, craniofacial, dental and skeletal abnormalities. Although genome-wide promoter analysis has revealed the existence of multiple TFII-I binding sites in embryonic stem cells (ESCs), there was no correlation between TFII-I occupancy and gene expression. Surprisingly, TFII-I recognizes the promoter sequences enriched for H3K4me3/K27me3 bivalent domain, an epigenetic signature of developmentally important genes. Moreover, we discovered significant differences in the association between TFII-I and BEN with the *cis*-regulatory elements in ESCs and embryonic craniofacial tissues. Our data indicate that in embryonic tissues BEN, but not the highly homologous TFII-I, is primarily recruited to target gene promoters. We propose a “feed-forward model” of gene regulation to explain the specificity of promoter recognition by TFII-I factors in eukaryotic cells.

## Introduction

TFII-I and BEN, products of paralogous genes *Gtf2i* and *Gtf2ird1*, display a dynamic expression pattern during mouse embryo development [1] [2]. At the blastocyst stage (E3.5–4.5) both transcription factors are localized to the cytoplasm and nuclei of the mouse inner cell mass (ICM) and trophectoderm. Consistently, in situ hybridization of mouse pre-implantation blastocysts revealed high levels of TFII-I mRNA in the ICM [3]. It is not surprising that the expression of TFII-I and BEN target genes was also detected in the early mouse embryo and ESCs [4] [5].

Although the TFII-I family members are expressed very early in development, they are not required for maintenance of pluripotency, because ablation of either *Gtf2i* or *Gtf2ird1* in mouse embryos does not lead to peri-implantation lethality [6]. The characteristic feature of TFII-I factors is a presence of multiple helix-loop-helix domains (I-repeats) that can serve as independent DNA-binding modules, although their chromatin recognition properties are still not fully understood. The SELEX procedure performed with a set of isolated I-repeats identified the core RGATTR sequence as a common DNA-binding motif for repeats 4 and 5 of BEN and for repeats 4 and 6 of TFII-I [7]. This core consensus sequence corresponds to the *bona fide* BEN-binding sites located in the upstream regulatory regions of *Hoxc8*, *Tnn1 Gsc, Scand3, Cfl, Shrm* and *Ezh2* genes [4] [8] [9] [10] [11] [12]. TFII-I and BEN bind to the DICE element TRTYBTCTHYACMR in the V_H_ promoters of IgH genes [13]. Furthermore, SELEX with the full-length BEN delineated a binding motif GGGRSCWGCGAYAGCCSSH that bears no sequence similarity to the DICE or RGATTR core consensus sequence [14]. Although TFII-I and BEN recognize the same or similar motifs, only TFII-I, together with USF1/USF2 heterodimer, binds to the upstream *cis* element RBEIII (ACTGCTGA) necessary for transcription of Human Immunodeficiency Virus Type 1 [15]. In addition, TFII-I interacts with the E-boxes (CANNTG) and the pyrimidine-rich Initiator element (YYANWYY) [16]. It was speculated that TFII-I regulates *c-Fos* as well as the set of estrogen-responsive genes by recognizing the Initiator sequence [17] [18]. The regulation of *VEGFR-2* and β-globin genes, for example, occurs through the recruitment of TFII-I to the Initiator and E-box elements, respectively [19] [20]. We reported that in mouse ESCs TFII-I binds to the canonical R4 consensus in the promoters of *Cfdp1*, *Sec23a* and *Nsd1* [21].

Despite these findings, the direct *in vivo* targets of TFII-I factors are poorly defined. In the present work, we report genome-wide promoter mapping in mouse ESCs and embryonic craniofacial tissues. Chromatin immunoprecipitation (ChIP)-coupled DNA microarray analysis (ChIP-chip) revealed multiple TFII-I and BEN binding sites across the upstream regulatory regions of many developmental regulators. In addition to recognizing the previously defined consensus sequences, these proteins associate with the novel *cis-*regulatory elements. Collectively, our data indicate that chromatin recognition by TFII-I transcription factors is more complicated and diverse than previously considered.

## Results

### A Genome-wide Promoter Screen for TFII-I and BEN Binding Sites in Mouse ESCs and Embryonic Craniofacial Tissues

**Figure 1:**
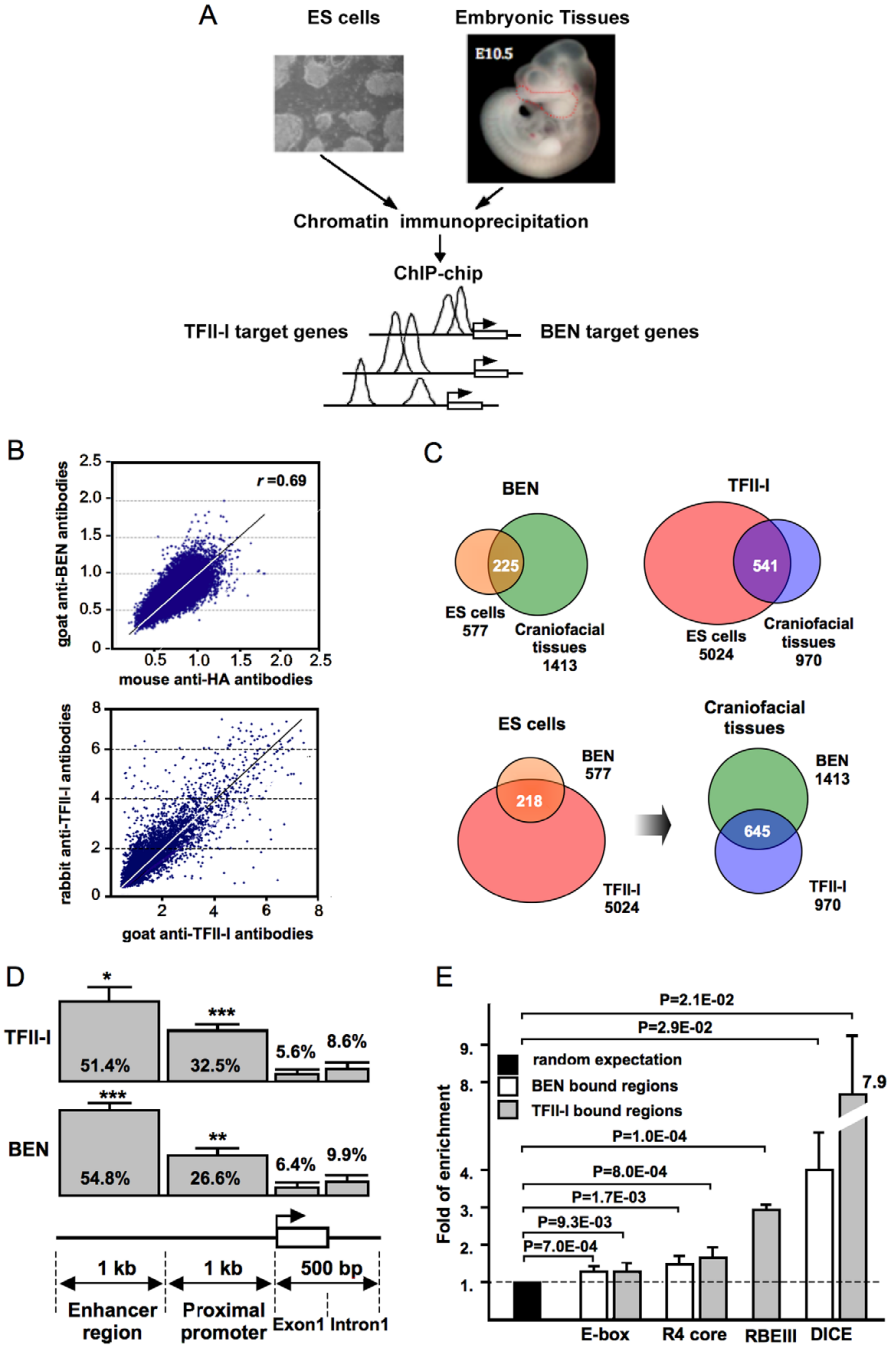
Chromatin isolation and ChIP-chip analysis. (A) The ChIP-chip strategy. Chromatin was isolated from mouse ESCs and the craniofacial region (CF) of E10.5 mouse embryos. Chromatin immunoprecipitation (ChIP) was performed with TFII-I and BEN-specific antibodies. CF region is marked in red with dashed lines. (B) The correlation between the genome-wide promoter binding using goat anti-BEN and mouse anti-HA antibodies to BEN (left) and rabbit and goat polyclonal antibodies to TFII-I (right). Spearman’s rank correlation coefficient (r) is calculated for each antibody pair. (C) The overall statistics of TFII-I and BEN target genes in ESCs and embryonic craniofacial tissues. (D) Distribution of the TFII-I and BEN-bound genomic sites with respect to the gene structure. The 2 kb region upstream of the transcription start site (TSS) is arbitrarily divided into two 1 kb segments (‘enhancer region’ and ‘proximal promoter’). 0.5 kb region downstream from TSS is split into exon and intron sequences. Bars represent the standard deviation calculated from four (TFII-I) or six (BEN) chip hybridizations, *p<0.1, **p<0.05, ***p<0.01.

The promoter ChIP-chip analysis was used to identify regions bound by TFII-I and BEN in the mouse ESC lines E14tg2a and Ainv15 and embryonic craniofacial tissues (ETs) derived from E10.5 mouse embryos (Fig. 1A). The chromatin immunoprecipitation (IP) was performed with two types of antibodies for each transcription factor: rabbit and goat polyclonal antibodies for TFII-I-bound chromatin IP and goat anti-BEN and mouse anti-HA antibodies BEN-bound chromatin IP. First, we have checked the consistency of ChIP-chip obtained with two different antibodies for each transcription factor. Spearman’s rank correlation coefficient (r = 0.7–0.9, Fig. 1B) determined for each antibody pair is comparable with technical replicates (r = 0.8–0.9, not shown). A positive correlation between experiments using different antibodies provides credibility to the TFII-I and BEN binding peaks.

5,744 TFII-I and 625 BEN binding peaks (FDR <0.19) were identified within 5,024 (Table S1) and 577 promoters (Table S2), respectively. 221 promoters are recognized by both transcription factors (Table S3A). Interestingly, TFII-I and BEN recognize the same sequence in 57 promoters (Table S3B).

We next investigated the occupancy of TFII-I factors in embryonic craniofacial tissues isolated from E10.5 mouse embryos. This experiment yielded 1,181 TFII-I and 1,520 BEN binding peaks (FDR <0.19) in 970 (Table S4) and 1,413 promoters (Table S5), respectively. Some peaks were detected in the promoter regions of the two closely located but oppositely directed genes and in such cases it was impossible to assign the binding peak to a corresponding gene. In contrast to the ESCs binding profile the promoter occupancy in ETs showed a 2.5-fold increase of BEN binding and a 5-fold decrease of TFII-I binding, respectively (Fig. 1C). Among 636 promoters occupied by both factors, 537 coincide with TFII-I and BEN binding to the same sequence (Table S6). Interestingly, the majority of genomic sites recognized by both proteins in ETs are also recognized by TFII-I in mouse ESCs. The density maps showed a high degree of TFII-I and BEN co-occupancy at the transcription start sites in ESCs and ETs (Fig. S1A).

### The DNA-binding Motifs Recognized by the TFII-I Family of Transcription Factors

Our next goal was to investigate a distribution of DNA-binding sites with respect to gene structure. CEAS software provides summary statistics including the percentages of peaks that reside in the proximal promoters (1 kb upstream from RefSeq 5′ start), 1st exon, 1st intron, and enhancer regions (>1 kb from RefSeq) [22]. A significant enrichment was detected in the proximal enhancers occupied by TFII-I and BEN in ESCs and in embryonic craniofacial tissues (Fig. 1*D*). Enrichment relative to random expectation (Fig. 1E) revealed a statistically significant abundance of the R4 consensus in the TFII-I (57.1%) and BEN-bound sequences (51.7%), respectively. E-boxes were the most enriched motifs and counted more than once among all known DNA-binding sequences. DICE is another sequence frequently bound by TFII-I and BEN. In contrast, RBEIII is a rare sequence recognized by TFII-I only.

Because we cannot rule out a probability that the chromatin recognition by the TFII-I family requires cooperation with additional transcription factors, DNA sequences around TFII-I and BEN bound sites were searched to identify potential protein partners listed in TRANSFAC and JASPAR databases. CEAS identified a number of hits for each motif both within the ChIP-regions and in the whole genome (p value <1.0E-5) (Tables S7 and S8). The genomic regions occupied by TFII-I and BEN showed significant enrichment for AP-2α, AP-2γ, ETF, Spz1, and E2F binding sequences. The BEN-bound sequences are also enriched with the recognition sites for SMADs, p300 and STATs. The relative abundance of these motifs suggests the existence of a specific protein interaction network.

The TFII-I and BEN bound sequences were also analyzed for the presence of new *cis*-regulatory elements [23]. We found three novel motifs with statistically significant enrichment (E-value less than 5E-21) (Fig. S1B and Table S9). TFII-I consensus sequence list is indicated in Fig. S1C.

**Figure 2:**
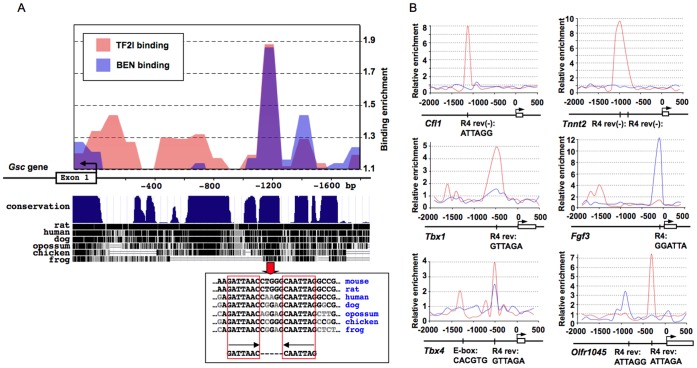
Genome-wide promoter recognition by TFII-I transcription factors in mouse ESCs. ( A) TFII-I and BEN bind to the distal element of the *Gsc* promoter. The conservation plot is downloaded from the UCSC genome browser (http://genome.ucsc.edu/). (B) TFII-I binds to the promoters of *Cfl1*, *Tnnt2* and *Tbx1* while BEN occupies the promoter of *Fgf3*. TFII-I and BEN recognize the same sequence in the *Tbx4* promoter although they bind to different *cis*-regulatory motifs within the promoter of *Olfr1045*. Red – TFII-I binding, blue – BEN binding.

It was previously reported that the TFII-I family interacts with the distal promoter element of *Gsc* [10] [11]. ChIP-chip confirmed TFII-I and BEN occupancy in the *Gsc* distal element corresponding to a stereotypical configuration of the two conserved R4 sequences (Fig. 2A). Multiple hits for E-boxes, R4 consensus, DICE and RBEIII elements were detected within the TFII-I and BEN occupied sites across a large set of developmentally regulated genes (Fig. 2B and Table S9). We noticed that each protein displayed a unique chromatin recognition mode. For example, TFII-I binding occurs at the more distal promoter sites of *Cfl1*, *Tnnt2* and *Tbx1* while BEN recognizes the more proximal promoter of *Fgf3* (Fig. 2B). Interestingly, TFII-I and BEN occupy the same sequence in the *Tbx4* promoter although they bind different *cis*-regulatory elements in the *Olfr1045* gene (Fig. 2B).

### Biological Functions of TFII-I and BEN Target Genes in Mouse ESCs

To gain insights into the TFII-I controlled biological processes we carried out the gene ontology (GO) analysis using the DAVID software [24]. The GO classification indicated significant enrichment in the ‘chromatin assembly’ (Fig. S2A and Table S10) and the ‘cell fate commitment’ categories amongst the TFII-I and BEN target genes in mouse ESCs (Fig. S2B and Table S10). Other over-represented cellular functions include ‘signal transduction’ for genes bound by TFII-I and ‘cell-cell signaling’ and ‘cytoskeleton organization’ for BEN targets. Multiple over-represented signaling pathways were found among TFII-I bound genes. BEN, on the other hand, primes mainly genes linked to the WNT signaling pathway (Fig. S2 *C* and Table S10).

### Biological Functions of TFII-I and BEN Target Genes in Embryonic Tissues

We also observed dramatic differences in the composition of genes targeted by TFII-I in mouse ESCs and ETs with regard to their biological functions. The most enriched cellular function among TFII-I bound genes in embryonic tissues is ‘signal transduction’ owing to a large group of olfactory, vomeronasal and taste receptor genes (Fig. S3B and Table S11). At the same time, BEN targets a broad spectrum of genes associated with ‘cell division’, ‘regulation of apoptosis’, ‘cell fate commitment’, ‘cell differentiation’, ‘cell motility’, ‘regulation of transcription’, ‘RNA processing’ and ‘regulation of translation’ (Supplemental Fig. S3A and Table S11). Many of these genes are linked to specific developmental processes, especially brain and skeletal development (Fig. S3C and Table S11).

### Genome-wide Promoter Occupancy by TFII-I and BEN does not Correlate with the Expression Levels of Target Genes in Mouse ESCs

We addressed the question whether the genomic sites occupied by TFII-I exert transcriptional regulatory activity. We analyzed the Affymetrix expression data (GEO database: GDS1616 record) derived from the same E14tg2a mouse ESC line used for ChIP-chip. The default P-value cut-offs (0.04 and 0.06) provide boundaries for defining Present, Marginal, or Absent calls. Absent indicates that the expression level is below the threshold of detection and close to zero. Marginal call indicates the cases of an uncertainty. The distribution of gene targets marked as absent, marginal and present is 49.1%:2.4%:48.5% for BEN and 48.7%:2.6%:48.7% for TFII-I, respectively. This observation clearly indicates that binding of TFII-I factors does not correlate with the transcriptional activity of the corresponding genes. Quantitative mRNA expression analysis from mouse ESCs [25] was used to compare transcription of target genes. Similarly, we failed to detect significant differences in the overall level of expression except the modest decrease in transcription among BEN bound genes.

### Correlation between TFII-I Target Genes and Embryonic Developmental Pathways

We analyzed genes bound by TFII-I in mouse ESCs and embryonic craniofacial tissues for various developmental processes. For this purpose the ChIP-chip gene list was compared with four different gene groups: 366 human genes associated with neural tube closure defects [26], 497 mouse genes linked to craniofacial development, 739 mouse genes associated with skeletal development and 976 mouse genes involved in brain development, respectively (MGI, http://www.informatics.jax.org/phenotypes.shtml). A chi-squared test was performed to estimate the average deviation of actually observed genes from the expected value of random variables (Table S12). We discovered that TFII-I binding sites are significantly enriched in ESCs, which is contrary to the enrichment of BEN in embryonic craniofacial tissues.

### Abrogation of TFII-I and BEN by siRNA Knockdown in Mouse Neural Crest Cells

To find a correlation between TFII-I and BEN promoter binding in ESCs and the expression of key developmental genes at later stages of differentiation, we investigated siRNA-mediated silencing of *Gtf2i* and *Gtf2ird1* in mouse neural crest cells. For this purpose we used the neural crest-derived JoMa1.3 cell line, which retains some features of stemness and the ability to differentiate into neurons, glia, smooth muscle cells, melanocytes and chondrocytes [27].

**Figure 3:**
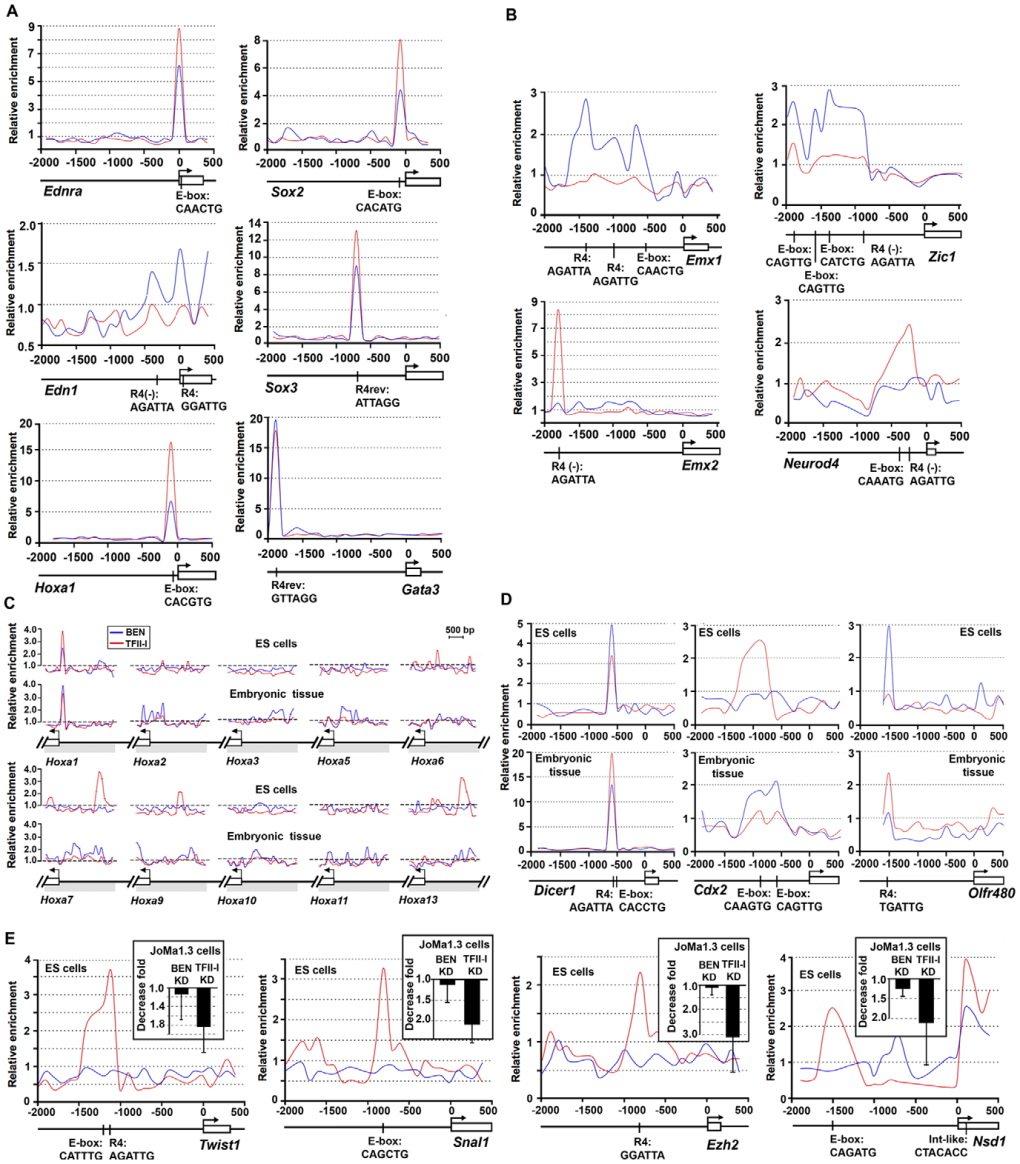
TFII-I factors occupy the promoters of key developmental regulators in ESCs and embryonic craniofacial tissues (ETs). (A) TFII-I and BEN bind to the promoters of *Ednra, Edn1, Sox2, Sox3, Hoxa1* and *Gata3* implicated in neural crest and craniofacial development. (B) TFII-I occupies the promoters of *Emx1, Emx2, Zic1* and *Neurod4* involved in brain development. The notation and labeling are as in Figure 2B. (C) TFII-I and BEN occupy the promoter regions of the *HoxA* cluster (*Hoxa1, Hoxa7* and *Hoxa13*) in mouse ESCs and ETs. (D) TFII-I and BEN recognize the same *cis*-regulatory element in the promoters of *Dicer, Cdx2* and *Olfr480* in stem cells and embryonic tissues. (E) TFII-I binds to the promoters of *Twist1, Snail2, Ezh2 and Nsd1* (red lines) in ESCs, although BEN does not bind to these promoters (blue lines). siRNA-mediated knockdown of TFII-I down-regulates expression of *Twist*, *Snail1, Ezh2* and *Nsd1* in embryonic neural crest cells (JoMa1.3 line). Error bars represent the standard deviation calculated from three independent knockdown experiments.

Three independent *Gtf2i* and *Gtf2ird1* siRNA-mediated knockdowns have been performed with JoMa1.3 cells. The knockdown effect was monitored at three different time intervals (9 h, 24 h and 72 h) post transfection. Average 9.8-fold decrease in *Gtf2i* expression (>90% knockdown) and 5.8-fold decrease in *Gtf2ird1* expression (∼80% knockdown) was observed 9 h post transfection. This silencing effect persisted through day 3 with the remaining 2.5-fold decrease of *Gtf2i* and 5-fold decrease of *Gtf2ird1* expression, respectively (Fig. S4). We observed no notable morphological changes in JoMa1.3 cells, although expression of neural crest markers *Twist* and *Snail1* as well as histone methyltransferases *Ezh2* and *Nsd1* was affected after TFII-I knockdown but not after BEN inactivation (Fig. 3E). ChIP-chip has also confirmed our previous observation that TFII-I occupies *Ezh2* and *Nsd1* proximal promoters in mouse ESCs [21]. Collectively, these results indicate that during ESC differentiation TFII-I regulates genes involved in cell fate commitment and lineage specification.

### Genome-wide Promoter Occupancy by TFII-I and BEN in ESCs and Embryonic Craniofacial Tissues

**Figure 4:**
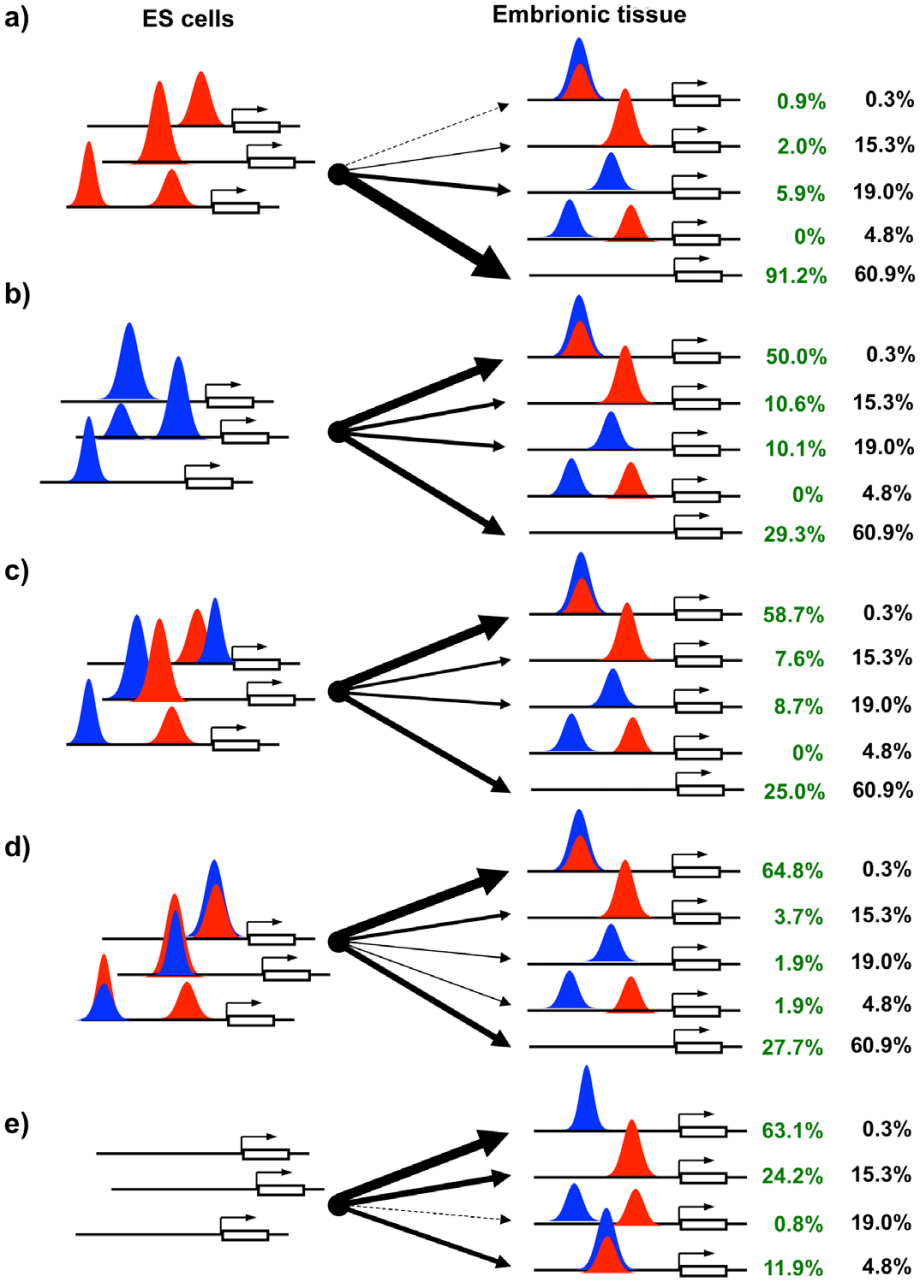
Promoter recognition by the TFII-I family. TFII-I (in red) and BEN (in blue) possess distinct promoter recognition properties in ESCs and embryonic craniofacial tissues (ETs). First, the majority of ESC promoters occupied by TFII-I become vacant in ETs (a); second, a large number of ESC promoters recognized by BEN recruit both transcription factors to the same site in ETs (b); third, the ESC promoters occupied by TFII-I and BEN are still recognized by both transcription factors in ETs, predominantly in the same sequence, although some sites lost their binding completely (d); and fourth, the promoters active in ETs recruit more BEN than TFII-I (e). The black numbers on the right indicate percentage expected from the random distribution of TFII-I or BEN binding. The green numbers indicate the observed distribution significantly deviated from the random distribution (chi-squared test).

We established that promoter recognition by TFII-I and BEN shows significant difference in ESCs and embryonic tissues. First, the majority of promoters occupied by TFII-I in ESCs become free in ETs (Fig. 4,a); second, a large number of ESC promoters bound by BEN recruit TFII-I and BEN to the same site in ET promoters (Fig. 4,b); third, ESC promoters occupied by TFII-I and BEN are still recognized by both transcription factors in ETs, predominantly at the same sites, although in some *cis*-regulatory sites TFII-I and BEN binding is lost completely (Fig. 4,c,d); and fourth, promoters active only in ETs recruit more BEN than TFII-I (Fig. 4,e). For example, both proteins occupy the same sequence in the *Hoxa1* and *Dicer* promoters, while BEN either replaces or is being replaced with TFII-I in the *Cdx2* and *Olfr480* promoters, respectively (Fig. 3C,D).

### Correlation between TFII-I and BEN Promoter Occupancy and Genome-wide Distribution of H3K27me3 and H3K4me3 Bivalent Marks

**Figure 5:**
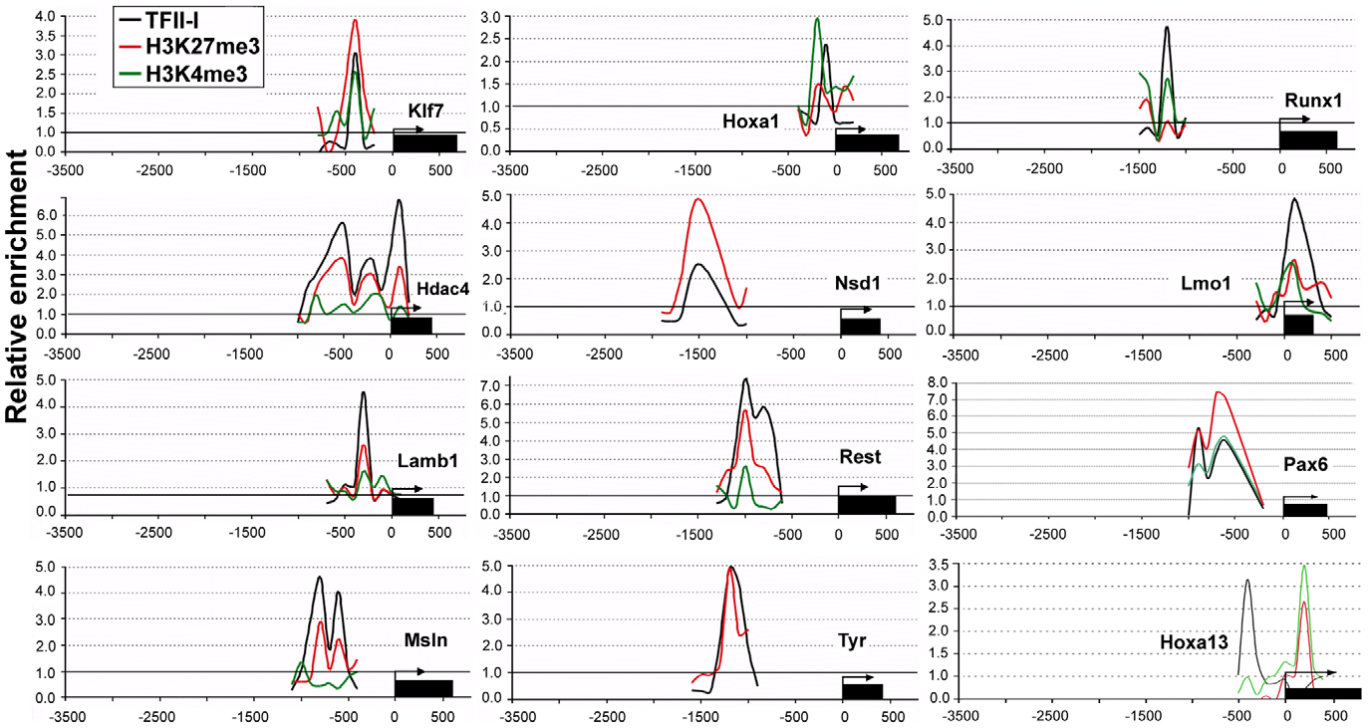
Colocalization of bivalent chromatin with TFII-I bound sites. TFII-I associates with the promoter regions of key developmental genes enriched for H3K4me3 and H3K27me3 marks.

**Figure 6:**
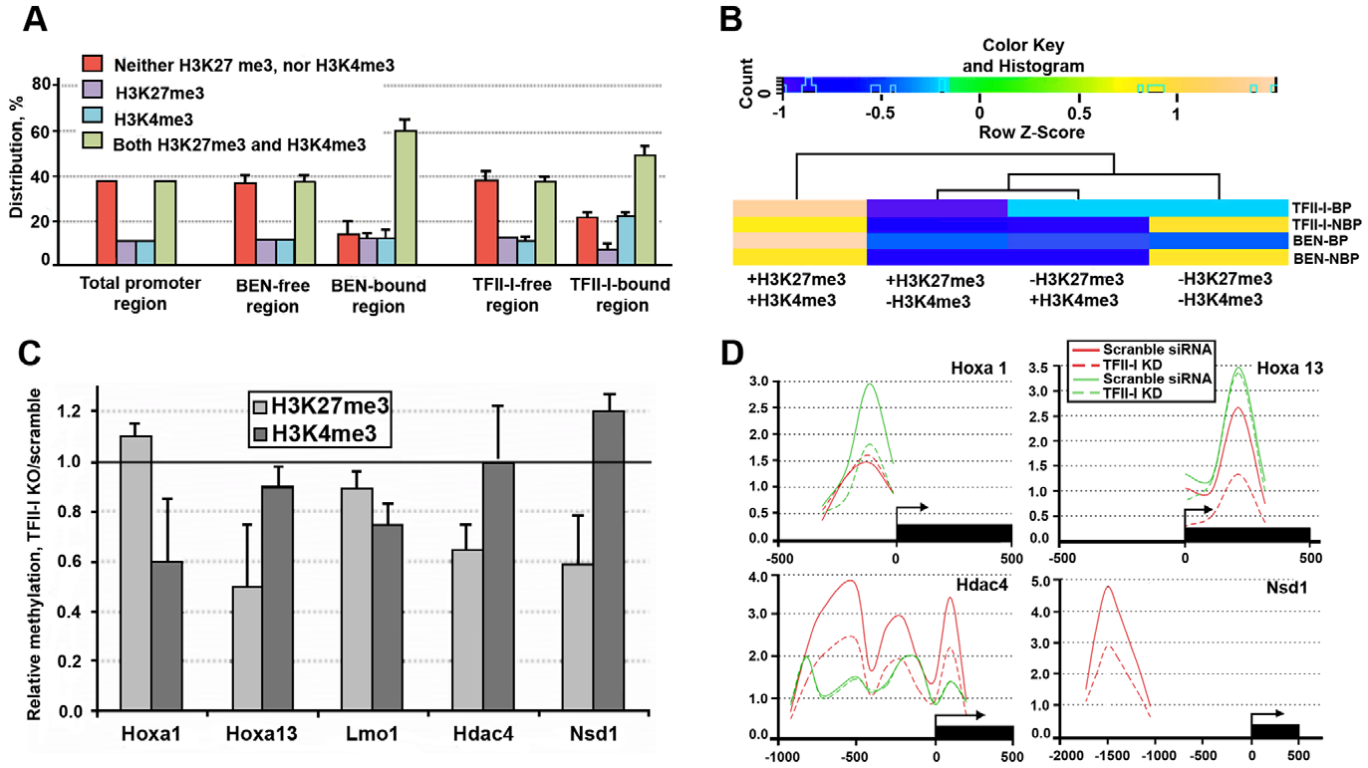
TFII-I binding overlaps with bivalent domain in ESCs. ( A) The promoter occupancy by TFII-I and BEN correlates with genome-wide distribution of H3K27me3 and H3K4me3 bivalent marks (green bars). (B) Heat map indicates the co-localization frequency of TFII-I and BEN with bivalent domain. TF2I-BP, TFII-I bound promoters; TF2I-NBP, promoters free of TFII-I; BEN-BP, BEN bound promoters; BEN-NBP, promoters free of BEN. (C) Depletion of TFII-I by siRNA knockdown reduces H3K4me3 at *Hoxa1* and H3K27me3 at *Hoxa13, Hdac4* and *Nsd1*. (D) ChIP revealed that TFII-I depletion affects H3K27me3 at the promoters of *Hoxa13, Hdac3* and *Nsd1* and H3K4me3 at the *Hoxa1* promoter. H3K27m3 is in red; H3K4me3 is in green.

The lack of correlation between promoter recognition by TFII-I and BEN and gene expression in mouse ESCs prompted us to examine the status of histone methylation in the genomic regions occupied by these factors. For this purpose we used H3K27me3 and H3K4me3 promoter-binding profile (NCBI GEO database: record GSE17387) performed on the same GPL8943 platform (mouse 385 K RefSeq Promoter Array) in B6D2F2 mouse ESCs [28]. We observed co-occupancy of H3K4me3/K27me3 bivalent marks and TFII-I factors in the promoters of many developmental regulators. The promoter occupancy for a few genes is illustrated in Fig. 5. Correlation analysis showed enrichment of bivalent marks (P<0.05) in the regulatory regions occupied by TFII-I or BEN (Fig. 6A). To confirm this observation, we used previously published ChIP-seq data sets for H3K4me3 and H3K27me3 (GSM307618 and GSM307619). The peak finding program MACS produced 26,339 H3K4me3 and 5,529 H3K27me3 peaks, respectively. Colocalization of bivalent domains with the genomic sites bound TFII-I factors is represented as a heat-map (Fig. 6B). We found a statistically significant (P<0.05) association between bivalent domain and promoter sites occupied by TFII-I and BEN. H3K27 tri-methylation at the promoters of *Hoxa13*, *Hdac4* and *Nsd1* (Fig. 6C,D) is significantly affected in cells depleted of TFII-I. We noticed that H3K4 tri-methylation at the *Hoxa1* promoter was also reduced in ESCs.

## Discussion

### Genome-wide Promoter Binding by TFII-I and BEN in ESCs and Embryonic Craniofacial Tissues

**Figure 7:**
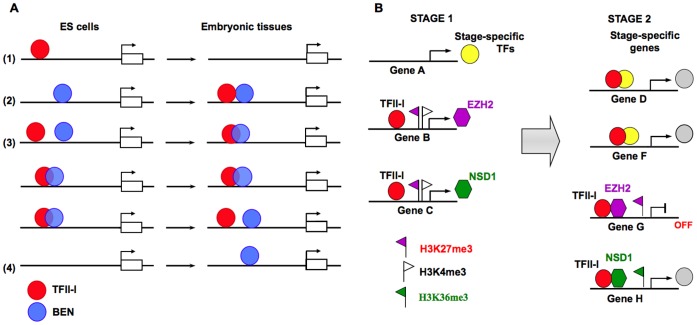
Target recognition by TFII-I factors. ( A) Chromatin recognition by TFII-I transcription factors in ESCs and embryonic craniofacial tissues (ETs). (1), the majority of TFII-I-bound ESC promoters tend to lose TFII-I binding in ETs. (2), the majority of BEN-bound ESC promoters recruit TFII-I de novo in ETs. (3), most of TFII-I and BEN-bound ESC promoters continue to retain these factors in ETs. (4), the promoters active in ETs recruit more BEN than TFII-I. (B) The feed-forward model explains the lack of correlation between the promoter binding by TFII-I and expression of the corresponding genes (stage 1, ESCs). Transcription factors (TFs) activated by TFII-I at stage 2 (ETs) can also recognize the TFII-I target sites and together they could initiate expression of stage-specific genes and additionally activate chromatin-modifying genes *Ezh2* and *Nsd1*. These epigenetic factors mark novel target promoters (genes F and G) for repression or activation (e.g. repressive mark H3K27me3 and active mark H3K36me3). Stage 1, embryonic stem cells; stage 2, embryonic tissues.

Our studies revealed that TFII-I and BEN recognize multiple distinct sequence motifs. In ESCs predominantly TFII-I primes the majority of genes associated with signal transduction and cell fate commitment. On a contrary, in embryonic craniofacial tissues BEN binds genes linked to tissue and organ development (Fig. S2, S3 and Table S10–11). We discovered that promoter occupancy by TFII-I and BEN is significantly different in ESCs and ETs. The following general rules were deduced from the promoter occupancy profiles (Fig. 7A). First, the majority of ESC promoters bound by TFII-I become vacant in ETs. Second, most ESC promoters recognized by BEN retain the ability to recruit both transcription factors in ETs. Third, the majority ESC promoters occupied by TFII-I and BEN recruit these factors to the same *cis*-regulatory sites in ETs. Fourth, BEN predominantly recognizes promoters active in ETs.

The under-representation of genes targeted by TFII-I in embryonic craniofacial tissues is in a strike contrast to the abundance of genes recognized by BEN (Table S4–12). In part, this could be explained by posttranslational modifications mediated by various signaling mechanisms that could alter the TFII-I binding ability as it was demonstrated for the TGFβ/activin-dependent induction of *Gsc* [11] [29].

### Diversity of the TFII-I Recognition Elements

ChIP-chip revealed that TFII-I and BEN display a complex DNA binding mode. Although, in the majority of cases, TFII-I family members bind to the canonical R4 consensus sequence or E-boxes, not all the high-confident binding sites can be explained by the presence of these motifs. MEME analysis has revealed that TFII-I factors recognize additional sequences (Fig. S1B and Table S9), but even these novel motifs cannot explain the full complexity of promoter occupancy. Nevertheless, we were able to confirm TFII-I and BEN binding to a previously characterized distal element of the *Gsc* promoter (Fig. 2A). Both proteins co-occupy the same site in the *Hoxa1* and *Dicer1* promoters in ESCs and embryonic tissues (Fig. 3C,D). TFII-I factors can also exhibit mutually exclusive DNA-binding mode. In mouse ESCs TFII-I and BEN colocalize at the promoters of *Olfr480* and *Cdx2*. In embryonic craniofacial tissues only TFII-I binds to the promoter of *Olfr480* while BEN replaces TFII-I in the *Cdx2* promoter (Fig. 3D).

Based on the sequence similarity the core consensus sequence RGATTR can be considered as a subset of the Initiator motif. Although the Initiator-like motifs were found among the TFII-I-bound sites, these sequences scatter frequently across the mammalian genome without specific protein binding capacity. The fact that not all the consensus sequences were identified at genomic loci bound by TFII-I and BEN suggests that promoter recognition is also determined by other transcription factors or the local chromatin structure. In this connection, it is notable that TFII-I or BEN binding sites coincide with the regions of extensive sequence conservation suggesting that additional regulatory proteins may influence their recruitment (Tables S7 and S8).

In conclusion, TFII-I factors display complex chromatin recognition, which differs from simple cooperative interaction or competition between TFII-I and BEN for a common binding site.

### Diversity of the TFII-I and BEN Target Genes

TFII-I and BEN prime a large group of the olfactory, vomeronasal, taste receptor and histone genes (Tables S1–6). The odorant receptor genes with over thousand members constitute the largest gene family in the mouse genome [30]–[31]. A mature olfactory sensory neuron is thought to express just one olfactory receptor. The transcriptional mechanisms and the regulatory elements that mediate the expression of a single olfactory receptor gene per neuron remain poorly understood [32]. BEN was previously identified as a transcription factor involved in the regulation of the olfactory receptor gene 262 [33] Among other abundant TFII-I and BEN targets are histone 1H1, 1H2 and 2H clusters (Tables S1–S6).

### The “Feed-forward Model” of TFII-I Regulation

Recruitment of TFII-I to a large number of developmental promoters does not correlate with the expression of the corresponding genes. Surprisingly, we discovered that TFII-I-bound regions co-localize with a bivalent chromatin structure associated with cell fate commitment and differentiation (Fig. 5,6). We speculate that TFII-I is involved in the formation or maintenance of the bivalent chromatin state, although we cannot exclude the possibility that TFII-I recognizes H3K4me3/K27me3-enriched genes and furthermore, the developmental dynamics of TFII-I binding depend on the availability of such bivalent marks. We recently found that during ESC differentiation TFII-I modulates expression of a large group of regulatory genes involved in chromatin modifying and epigenetic reprogramming [5]. Collectively, these data prompted us to adopt the “feed-forward model” of gene regulation (Fig. 7B) previously proposed to explain the ability of transcription factors to recognize multiple genomic loci [34]. We have previously proposed that TFII-I may exert a specific regulatory effect upon the target genes by changing the histone methylation status of bivalent chromatin [35].

According to this model TFII-I binds to the promoters of bivalent genes with delayed expression except of a few developmental regulators (stage 1, stem cells) that together with TFII-I, regulate other genes (stage 2, embryonic tissues) (Fig. 7B). Among TFII-I targets are genes implicated in lineage differentiation (*Gsc*, members of *Hox* and *Dlx* family) and epigenetic modulators (*Ezh2* and *Nsd1* histone methyltransferase and *Hdac4* histone deacetylase).

## Materials and Methods

### ESC Maintenance and Differentiation

Mouse ESCs Ainv15 (SCRC-1029) and E14tg2a (CRL-1821) were obtained from ATCC, VA. Ainv15 was maintained on the feeder cells in DMEM (Invitrogen, CA) supplemented with 15% fetal calf serum (HyClone Laboratories, UT), 1000 U/ml LIF (Chemicon, CA), non-essential amino acids (Invitrogen, CA), penicillin-streptomycin-L-glutamine and 2-mercaptoethanol. The formation of embryoid bodies was initiated in E14tg2a cells by removal of LIF and 2-mercaptoethanol. After depletion of the feeder cells, 5×10^5^ ESCs were allowed to aggregate in suspension culture in Petri dishes. E14tg2a cells were grown in a feeder-free environment in KD MEM (Gibco, CA) containing 15% knockout serum replacement (Invitrogen, CA), 1000 U/ml LIF (Chemicon, CA), 0.1 mM non-essential amino acids, penicillin-streptomycin-L-glutamine and 2-mercaptoethanol. The cell envelope-associated alkaline phosphatase (ALP) activity was determined using a commercially available kit ALP-10 (Sigma-Aldrich, MO). Briefly, a test cell population was washed twice in PBS, lysed in 0.1% Triton X for 5 min prior to addition of the p-nitrophenyl phosphate buffer.

### Culture of JoMa1.3 Cells and siRNA Knockdown of TFII-I Proteins

Mouse neural crest cell line JoMa1.3 [27] was grown on cell culture dishes coated with fibronectin. For maintenance, DMEM:Ham’s F12 (1:1) medium (Invitrogen, CA) was supplemented with 1% N2-Supplement (Invitrogen, CA), 2% B27-Supplement (Invitrogen, CA), 10 ng/ml EGF (R&D Systems Inc., MN), 1 ng/ml FGF (R&D Systems Inc., MN), 100 U/ml Penicilin–Streptomycin (Invitrogen, CA), 200 nM Tamoxifen (Sigma-Aldrich, MO) and 10% chick embryo extract (Gemini Bio-Products, CA). siRNA-mediated knockdown of *Gtf2i* or *Gtf2ird1* in JoMa1.3 cells was performed using ON-TARGETplus SMARTpool siRNA (Dharmacon, IL). Three independent transfections were performed for each gene. To ensure minimal non-specific effects on gene expression we used the control negative siRNA (ON-TARGETplus Non-targeting Pool).

### Generation of Tet-inducible BEN Expressing ESC Line

Murine full-length cDNA for *Gtf2ird1* (GenBank accession #AY030288) was amplified by PCR using Herculase II DNA polymerase (Stratagene, CA). The PCR product was cloned into pLox vector and resulting construct plox/Gtf2ird1 was co-electroporated with pSalk-Cre into the genetically modified for this purpose cell line Ainv15 [36]. After electroporation stable clones were obtained by selection with 350 µg/ml of G-418 (Gibco, CA), and screened for integration by PCR. Cells were induced for BEN expression with 1 mg/ml doxycycline (Sigma-Aldrich, MO) in media without LIF.

### QRT-PCR Analysis

cDNA was synthesized from 0.1–2 µg of RNA with Omniscript Reverse transcription kit (Qiagen, CA) using oligo dT or random N_12_ primers. Quantitative RT-PCR was performed on Applied Biosystems 7300 or on Bio-Rad MiniOpticon MJ Real-Time PCR system using TaqMan expression assays (Applied Biosystems, CA). The list of assays used in this study is given in Supplemental table S1. PCR reactions were performed in duplicates for each cDNA sample. All transcript levels were normalized to that of *Gapdh* and the relative expression ratios were calculated using the delta-delta Ct method [37] or the Pfaffl method [38], when probe efficiency was lower than 100%. Statistical analyses were performed using the InStat 3.0b software (GraphPad Software Inc., CA).

### Chromatin Immunoprecipitation (ChIP) and ChIP-chip

ChIP experiments were performed with mouse ESC lines E14tg2a and Ainv15 or embryonic tissues using ChIP-IT kit (Active Motif, CA). About 120 mouse embryos on gestational day 10.5 (E10.5) were dissected by a tungsten needle to produce craniofacial embryonic material for ChIP. Chromatin samples were sheared using Branson sonifier 150 (Heinemann, Germany). Chromatin-binding proteins were reverse cross-linked and digested with Proteinase K (Thermo Fisher Scientific, MA). 10 µl of the chromatin supernatant was saved as Input DNA before immunoprecipitation (IP). ChIP experiment was performed with goat anti-BEN polyclonal IgG M-19, goat anti-TFII-I polyclonal IgG V-18, mouse anti-HA monoclonal IgG2b 12CA5 (sc-14714X, sc-9943X and sc-57592, Santa Cruz Biotechnology, CA) and with rabbit anti-TFII-I polyclonal antibodies 4562 (Cell Signaling Technology, MA) and antibody-chromatin complexes were purified using protein G-coated magnetic beads (Active Motif, CA). After reverse cross-linking and purification on QIAquick spin columns (Quagen, CA), immunoaffinity-enriched DNA fragments (IP) and the input samples were amplified using whole-genome GenomePlex Complete WGA kit (Sigma-Aldrich, MO). IP and input samples were labeled in separate reactions with Cy5 and Cy3, respectively, and then were co-hybridized to Mouse 385 K RefSeq Promoter Arrays (Roche NimbleGen, WI). The data were extracted using NimbleScan software. Peaks were detected by searching for four or more probes with a signal above a cut-off value using a 500-bp sliding window. A log_2_ ratio of the IP versus input samples was calculated and the transcription start site mapping was performed using SignalMap software (Roche NimbleGen, WI). The promoter recruitment was validated experimentally by independent ChIP.

### Histone Methylation Analysis

For analysis of histone methylation, siRNA-mediated knockdown of *Gtf2i* or *Gtf2ird1* was performed in mouse ESCs E14tg2a using ON-TARGETplus SMARTpool siRNAs (Dharmacon, IL). Anti-H3K27me3 (39535, Active Motif, CA), anti-H3K4me3 (ab1012, Abcam, MA), and anti-H3 (39163, Active Motif, CA) antibodies were used for ChIP experiment. Quantitative data were obtained by qRT-PCR with SYBR Green and custom-made primers and expressed as a ratio of methylated H3 versus total H3 histone. For co-locaization analysis, H3K4me3 and H3K27me3 ChIP-Seq data were downloaded from NCBI GEO (GSM307618 and GSM307619).

### Bioinformatics and Statistical Analysis

The putative TFII-I and BEN binding sites were searched as a pattern consensus on both strands using the MacVector 9.5 software (Oxford Molecular Group, UK). Histone peaks were determined using the peak finding program MACS (Model-based Analysis for ChIP-Seq) from Dana-Farber Cancer Institute and Harvard School of Public Health in Boston, MA (http://liulab.dfci.harvard.edu/MACS/). The TFII-I-binding peaks and methylated regions were considered co-localized if they overlapped or the distance between the closer ends of the two genomic coordinates was less than 200 bp apart from each other. The numbers for the co-localization between all different combinations were converted into percent ratios and used Heatmap2 (http://hosho.ees.hokudai.ac.jp/~kubo/Rdoc/library/gplots/html/heatmap.2.html) package in R to produce the heat-map visualization. Heatmap represents the numbers in color code chart with different colors used to represent different z-scores for the data. To compare DNA sequences the EMBOSS Pairwise Alignment Tools were used (www.ebi.ac.uk/tools/emboss/align/index.html). Statistical analysis was performed with InStat 3.0b (GraphPad Software Inc., CA).

## Supporting Information

Figure S1 **Density map and novel TFII-I binding consensus sequences. (**A) Colocalization of TFII-I binding in mouse ESCs (a) and BEN binding in embryonic craniofacial tissues (b). For the density map, 5744 TFII-I-binding peaks from ESCs (a) and 1520 BEN-binding peaks from embryonic craniofacial tissues (b) were aligned relative to transcription start sites (left panels) and the corresponding BEN-binding peaks (a) or TFII-I-binding peaks (b) are shown in the right panel. Red dashed lines indicate position of the aligned “reference” peaks. (B) The novel consensus sequences recognized by TFII-I factors. (C) The canonical TFII-I and BEN binding motifs.
(DOC)

Figure S2 **The gene ontology and KEGG pathway analysis in mouse ESCs.** (A) The basic cellular functions. **(**B) The basic cellular processes. (C) The KEGG pathways. The red and blue bars represent fold enrichment of the TFII-I and BEN bound genes, respectively. The red stars indicate the statistically significant functional categories (p-value 1.5E-1).
(DOC)

Figure S3 **Gene ontology and KEGG pathway analysis of TFII-I target genes in mouse embryonic craniofacial tissues.** (A) The basic cellular functions. (B) The basic cellular processes. (C) The developmental categories.
(DOC)

Figure S4 **siRNA knockdown efficiency of TFII-I and BEN in JoMa cells.**
(DOC)

Table S1 **TFII-I binding sites in the ESC promoters.**
(XLS)

Table S2 **BEN binding sites in the ESC promoters.**
(XLS)

Table S3 **Promoters in ESCs recognized by TFII-I factors.**
(XLS)

Table S4 **Promoters in embryonic craniofacial tissues bound by TFII-I.**
(XLS)

Table S5 **Promoters in embryonic craniofacial tissues bound by BEN.**
(XLS)

Table S6 **Promoters in ESCs recognized by TFII-I and BEN to the same sequence.**
(XLS)

Table S7 **Enrichment for transcription factor binding motifs across the TFII-I and BEN bound promoter regions in ESCs.**
(XLS)

Table S8 **Enrichment for transcription factor binding motifs in the TFII-I and BEN bound promoter regions in embryonic craniofacial tissues.**
(XLS)

Table S9 **The consensus binding motifs within the TFII-I and BEN bound promoter regions.**
(DOC)

Table S10 **Pathway analysis in mouse ESCs.**
(DOC)

Table S11 **Pathway analysis in mouse embryonic craniofacial tissues.**
(DOC)

Table S12 **TFII-I transcription factors target a large set of developmental regulators.**
(DOC)
