# Supplementary figures and images for: Diversity and Complexity in Chromatin Recognition by TFII-I Transcription Factors in Pluripotent Embryonic Stem Cells and Embryonic Tissues
Source: PLoS One. 2012 Sep 10;7(9):e44443. doi: 10.1371/journal.pone.0044443 (PMC3438194; doi:10.1371/journal.pone.0044443)

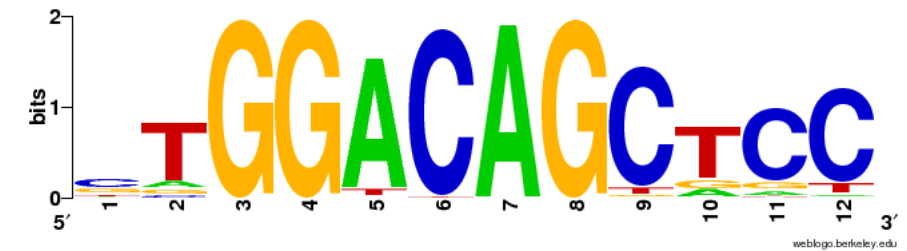

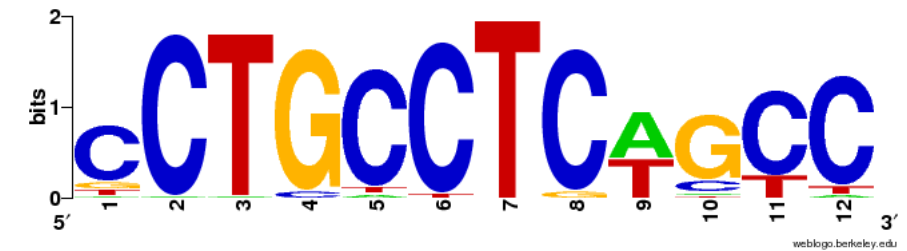

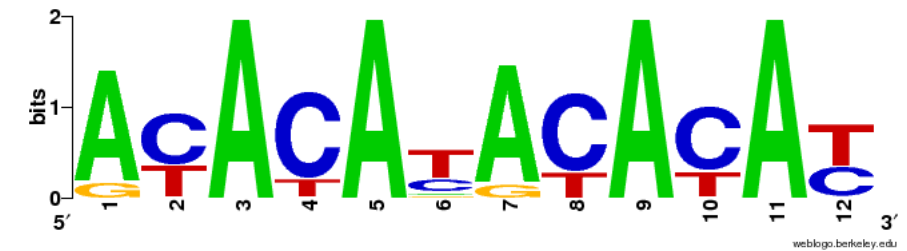


a

b


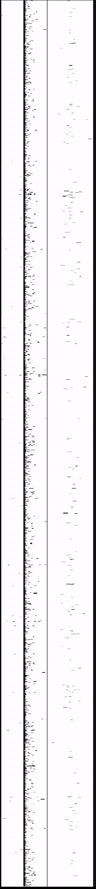


**TFII-I**

**BEN**

**-5 kb 0 5 kb**

**TFII-I**


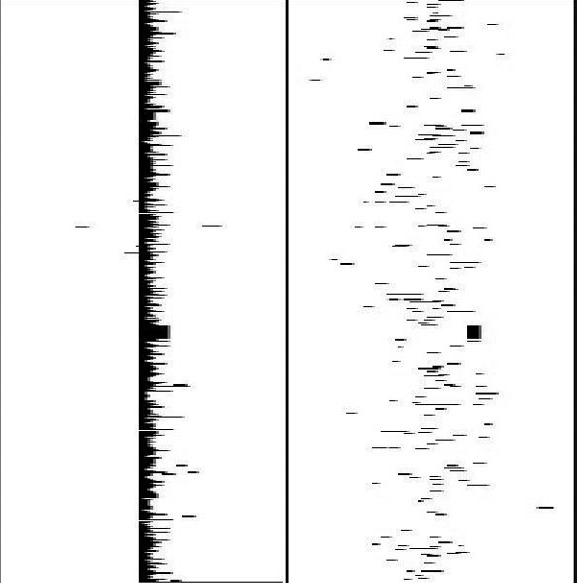


**BEN**

**-5 kb 0 5 kb**

**A**

**B**

**C**


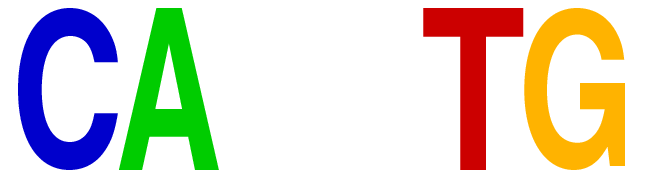

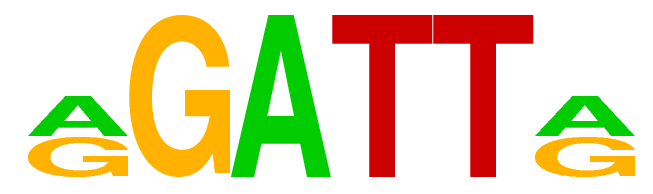

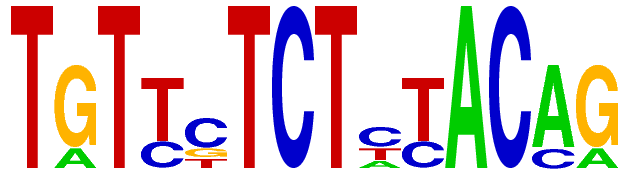

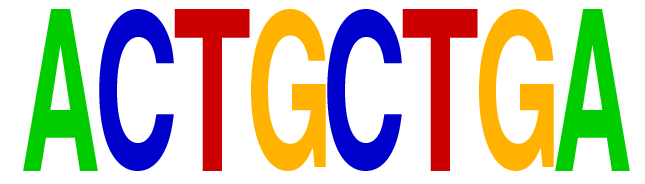


**E-box**

**R4 core**

**RBEIII**

**DICE**

Supplement: Figure S1 — Density map and novel TFII-I binding consensus sequences. (A) Colocalization of TFII-I binding in mouse ESCs (a) and BEN binding in embryonic craniofacial tissues (b). For the density map, 5744 TFII-I-binding peaks from ESCs (a) and 1520 BEN-binding peaks from embryonic craniofacial tissues (b) were aligned relative to transcription start sites (left panels) and the corresponding BEN-binding peaks (a) or TFII-I-binding peaks (b) are shown in the right panel. Red dashed lines indicate position of the aligned “reference” peaks. (B) The novel consensus sequences recognized by TFII-I factors. (C) The canonical TFII-I and BEN binding motifs. (DOC) [file pone.0044443.s001.doc]
