# Supplementary material for: Diversity and Complexity in Chromatin Recognition by TFII-I Transcription Factors in Pluripotent Embryonic Stem Cells and Embryonic Tissues
Source: PLoS One. 2012 Sep 10;7(9):e44443. doi: 10.1371/journal.pone.0044443 (PMC3438194; doi:10.1371/journal.pone.0044443)
Supplement: Figure S2 — The gene ontology and KEGG pathway analysis in mouse ESCs. (A) The basic cellular functions. (B) The basic cellular processes. (C) The KEGG pathways. The red and blue bars represent fold enrichment of the TFII-I and BEN bound genes, respectively. The red stars indicate the statistically significant functional categories (p-value 1.5E-1). (DOC) [file pone.0044443.s002.doc]

*

*

*

*

**4.4E-01**

**1.5E-01**

**1.1E-58**

**2.6E-01**

**8.6E-03**

**1.4E-01**

**1.6E-01**

*

*

*

*

*

*

*

*

*

**3.9E-02**

**2.4E-02**

**1.1E-02**

**7.2E-03**

**2.4E-02**

**4.1E-05**

**1.4E-02**

**4.9E-02**

**A**

# B

**C**

**5.9**

**1.8E-06**

*

*

**9.7E-04**

**2.3E-02**

*

*

**5.5E-02**
