# Supplementary material for: Diversity and Complexity in Chromatin Recognition by TFII-I Transcription Factors in Pluripotent Embryonic Stem Cells and Embryonic Tissues
Source: PLoS One. 2012 Sep 10;7(9):e44443. doi: 10.1371/journal.pone.0044443 (PMC3438194; doi:10.1371/journal.pone.0044443)
Supplement: Figure S3 — Gene ontology and KEGG pathway analysis of TFII-I target genes in mouse embryonic craniofacial tissues. (A) The basic cellular functions. (B) The basic cellular processes. (C) The developmental categories. (DOC) [file pone.0044443.s003.doc]

*

*

*

*

*

*

**1.1E-04**

**1.0E-02**

**1.2E-41**

**2.7E-14**

**1.3E-04**

**3.7E-36**

*

*

*

*

*

*

**1.3E-05**

**5.9E-05**

**6.9E-12**

**4.2E-20**

**1.3E-04**

**4.5E-115**

*

*

*

*

*

*

*

**1.9E-32**

**2.9E-13**

**3.1E-23**

**2.8E-28**

**1.5E-21**

**5.3E-11**

**1.9E-04**

**A**

**B**

# C
