# Supplementary material for: Diversity and Complexity in Chromatin Recognition by TFII-I Transcription Factors in Pluripotent Embryonic Stem Cells and Embryonic Tissues
Source: PLoS One. 2012 Sep 10;7(9):e44443. doi: 10.1371/journal.pone.0044443 (PMC3438194; doi:10.1371/journal.pone.0044443)
Supplement: Figure S4 — siRNA knockdown efficiency of TFII-I and BEN in JoMa cells. (DOC) [file pone.0044443.s004.doc]

Supplementary Figure 4. The effectiveness of siRNA KD in JoMa cells.

Experiment 1

**BEN**

**TFII-I**

**BEN**

**TFII-I**

Experiment 2

**BEN**

**TFII-I**

Experiment 3
