# Supplementary material for: Diversity and Complexity in Chromatin Recognition by TFII-I Transcription Factors in Pluripotent Embryonic Stem Cells and Embryonic Tissues
Source: PLoS One. 2012 Sep 10;7(9):e44443. doi: 10.1371/journal.pone.0044443 (PMC3438194; doi:10.1371/journal.pone.0044443)
Supplement: Table S9 — The consensus binding motifs within the TFII-I and BEN bound promoter regions. (DOC) [file pone.0044443.s013.doc]

Supplemental Table 9. % (± SEM) of consensus sequences within the TFII-I and BEN bound promoter regions.

|  | **TFII-I bound regions** | **BEN bound regions** |
| --- | --- | --- |
| **R4 core** | 57.1 ± 2.00 | 51.7 ± 3.02 |
| **E-box** | 174.6 ± 2.40 | 176.9 ± 2.53 |
| **DICE** | 0.19 ± 0.040 | 0.10 ± 0.024 |
| **RBEIII** | 1.59 ± 0.055 | N/A |
| **Novel TFII-I binding motifs** | | |
| **SDGGACAGYDVY** | 1.7 ± 0.04 | 1.7 ± 0.11 |
| **BCTGYCTSWSYY** | 3.7 ± 0.26 | 5.7 ± 0.64 |
| **RTAYAYRYAYAY** | 2.9 ± 0.57 | 2.6 ± 0.23 |
