# Supplementary material for: Diversity and Complexity in Chromatin Recognition by TFII-I Transcription Factors in Pluripotent Embryonic Stem Cells and Embryonic Tissues
Source: PLoS One. 2012 Sep 10;7(9):e44443. doi: 10.1371/journal.pone.0044443 (PMC3438194; doi:10.1371/journal.pone.0044443)
Supplement: Table S10 — Pathway analysis in mouse ESCs. (DOC) [file pone.0044443.s014.doc]

Supplemental Table 10. GO analysis in ES cells.

A. GO analysis in ES cells.

|  | Count | % of genes | Fold Enrichment | P value | Count | % of genes | Fold Enrichment | P value |
| --- | --- | --- | --- | --- | --- | --- | --- | --- |
|  | BEN target genes | | | | TFII-I target genes | | | |
|  |  |  |  |  |  |  |  |  |
| GO:0031497~chromatin assembly | **13** | **2.16** | **5.9** | **1.8E-06** | **28** | **0.53** | **1.3** | **9.7E-04** |
| GO:0045449~regulation of transcription | **82** | **13.64** | **1.2** | **2.3E-02** | 797 | 11.38 | 0.9 | 3.6E-01 |
| GO:0006396~RNA processing | 7 | 1.16 | 0.5 | 9.9E-01 | 160 | 2.45 | 1.0 | 8.5E-04 |
| GO:0051049~regulation of transport | 7 | 1.16 | 0.8 | 8.5E-01 | **122** | **1.79** | **1.1** | **6.5E-03** |
| GO:0006417~regulation of translation | 3 | 0.50 | 1.0 | 8.0E-01 | **21** | **0.79** | **1.5** | **5.5E-02** |
| GO:0031399~regulation of protein modification | 0 | 0.00 |  |  | 68 | 0.98 | 1.1 | 2.7E-01 |
| GO:0019222~regulation of metabolic process | 98 | 16.31 | 1.1 | 1.2E-01 | 1082 | 15.44 | 1.0 | 5.0E-01 |
|  |  |  |  |  |  |  |  |  |
| GO:0051301~cell division | 10 | 1.66 | 1.2 | 4.4E-01 | 111 | 1.59 | 1.0 | 2.8E-03 |
| GO:0042981~regulation of apoptosis | 15 | 2.50 | 0.9 | 7.6E-01 | 220 | 3.00 | 1.0 | 8.7E-09 |
| GO:0007267~cell-cell signaling | **13** | **2.16** | **1.5** | **1.5E-01** | 122 | 1.74 | 1.1 | 7.8E-04 |
| GO:0007165~signal transduction | 70 | 11.65 | 0.9 | 9.3E-01 | **916** | **19.96** | **1.3** | **1.1E-58** |
| GO:0007010~cytoskeleton organization | 13 | 2.16 | 1.4 | 2.6E-01 | 129 | 1.85 | 1.0 | 4.0E-01 |
| GO:0007155~cell adhesion | 18 | 3.00 | 1.1 | 4.8E-01 | **159** | **3.73** | **1.2** | **8.6E-03** |
| GO:0045165~cell fate commitment | **8** | **1.33** | **1.8** | **1.4E-01** | **26** | **0.98** | **1.3** | **1.6E-01** |
| GO:0030154~cell differentiation | 44 | 7.32 | 0.9 | 7.2E-01 | 601 | 8.62 | 1.0 | 2.9E-04 |
| GO:0048870~cell motility | 6 | 1.00 | 0.7 | 9.2E-01 | 114 | 1.61 | 1.0 | 1.3E-02 |
|  |  |  |  |  |  |  |  |  |
| GO:0032502~developmental process | 86 | 14.31 | 1.1 | 3.2E-01 | 1060 | 15.21 | 1.0 | 5.4E-07 |
| GO:0009888~tissue development | 22 | 3.66 | 1.2 | 3.3E-01 | 151 | 3.57 | 1.0 | 2.3E-01 |
| GO:0048513~organ development | 54 | 8.99 | 1.1 | 2.8E-01 | 652 | 9.31 | 1.0 | 2.2E-06 |
| GO:0007399~nervous system development | 0 | 0.00 |  |  | 328 | 4.70 | 1.0 | 4.3E-03 |
| GO:0007420~brain development | 7 | 1.16 | 0.8 | 8.6E-01 | 121 | 1.74 | 1.1 | 4.6E-01 |
| GO:0048705~skeletal system morphogenesis | 0 | 0.00 |  |  | 55 | 0.81 | 1.1 | 4.2E-01 |
| GO:0007517~muscle organ development | 5 | 0.83 | 1.0 | 7.7E-01 | 70 | 0.97 | 1.0 | 5.5E-01 |

Table 2B. Pathways in ES cells.

|  | Count | % of genes | Fold Enrichment | P value | Count | % of genes | Fold Enrichment | P value |
| --- | --- | --- | --- | --- | --- | --- | --- | --- |
|  | BEN target genes | | | | TFII-I target genes | | | |
|  |  |  |  |  |  |  |  |  |
| P00060:Ubiquitin proteasome pathway | 0 | 0.00 |  |  | **16** | **0.62** | **2.0** | **1.1E-02** |
| mmu04310:Wnt signaling pathway | **6** | **1.00** | **1.5** | **3.9E-02** | **43** | **1.03** | **1.3** | **2.4E-02** |
| MAPKinase Signaling Pathway | 0 | 0.00 |  |  | 26 | 0.50 | 1.2 | 2.3E-03 |
| P00010:B cell activation | 0 | 0.00 |  |  | **36** | **0.54** | **1.3** | **7.2E-03** |
| P00053:T cell activation | 0 | 0.00 |  |  | 48 | 0.72 | 1.1 | 5.7E-02 |
| P04393:Ras Pathway | 0 | 0.00 |  |  | 22 | 0.42 | 1.2 | 8.0E-03 |
| P00021:FGF signaling pathway | 0 | 0.00 |  |  | **15** | **0.58** | **1.3** | **2.4E-02** |
| mmu04370:VEGF signaling pathway | 0 | 0.00 |  |  | **30** | **0.76** | **1.9** | **4.1E-05** |
| NF-kB Signaling Pathway | 0 | 0.00 |  |  | **8** | **0.31** | **2.9** | **1.4E-02** |
| P00052:TGF-beta signaling pathway | 0 | 0.00 |  |  | **34** | **1.28** | **1.4** | **4.9E-02** |
| P00048:PI3 kinase pathway | 0 | 0.00 |  |  | 22 | 0.83 | 1.1 | 3.6E-01 |
