# Supplementary material for: Diversity and Complexity in Chromatin Recognition by TFII-I Transcription Factors in Pluripotent Embryonic Stem Cells and Embryonic Tissues
Source: PLoS One. 2012 Sep 10;7(9):e44443. doi: 10.1371/journal.pone.0044443 (PMC3438194; doi:10.1371/journal.pone.0044443)
Supplement: Table S11 — Pathway analysis in mouse embryonic craniofacial tissues. (DOC) [file pone.0044443.s015.doc]

Supplemental Table 11.

A. GO analysis in embryonic craniofacial tissues.

|  | Count | % of genes | Fold Enrichment | P value | Count | % of genes | Fold Enrichment | P value |
| --- | --- | --- | --- | --- | --- | --- | --- | --- |
|  | BEN target genes | | | | TFII-I target genes | | | |
|  |  |  |  |  |  |  |  |  |
| GO:0031497~chromatin assembly | **17** | **0.74** | **1.7** | **1.0E-02** | **24** | **0.93** | **2.4** | **1.1E-04** |
| GO:0045449~regulation of transcription | **457** | **19.90** | **1.8** | **1.2E-41** | 223 | 8.68 | 0.7 | 1.0E-00 |
| GO:0006396~RNA processing | **107** | **4.66** | **2.1** | **2.7E-14** | 35 | 1.36 | 0.5 | 1.0E-00 |
| GO:0051049~regulation of transport | 29 | 1.26 | 0.9 | 0.8E-01 | 19 | 0.74 | 0.5 | 1.0E-00 |
| GO:0006417~regulation of translation | **26** | **1.13** | **2.3** | **1.3E-04** | 9 | 0.35 | 0.7 | 1.0E-00 |
| GO:0031399~regulation of protein modification | 0 | 0.00 |  |  | 9 | 0.35 | 0.4 | 1.0E-00 |
| GO:0019222~regulation of metabolic process | **542** | **23.60** | **1.6** | **3.7E-36** | 274 | 10.67 | 0.7 | 1.0E-00 |
|  |  |  |  |  |  |  |  |  |
| GO:0051301~cell division | **58** | **2.52** | **1.8** | **1.3E-05** | 23 | 0.89 | 0.6 | 1.0E-00 |
| GO:0042981~regulation of apoptosis | **95** | **4.14** | **1.5** | **5.9E-05** | 36 | 1.40 | 0.5 | 1.0E-00 |
| GO:0007267~cell-cell signaling | 40 | 1.74 | 1.2 | 1.7E-01 | 32 | 1.24 | 0.8 | 9.5E-01 |
| GO:0007165~signal transduction | 0 | 0.00 |  |  | **771** | **30.02** | **2.1** | **4.5E-115** |
| GO:0007010~cytoskeleton organization | 41 | 1.78 | 1.1 | 3.5E-01 | 20 | 0.78 | 0.5 | 1.0E-00 |
| GO:0007155~cell adhesion | 62 | 2.70 | 1.0 | 7.1E-01 | 79 | 3.08 | 1.0 | 4.6E-01 |
| GO:0045165~cell fate commitment | **49** | **2.13** | **2.9** | **6.9E-12** | 0 | 0.0 |  |  |
| GO:0030154~cell differentiation | **297** | **12.92** | **1.6** | **4.2E-20** | 129 | 5.02 | 0.6 | 1.0E-00 |
| GO:0048870~cell motility | **55** | **2.39** | **1.7** | **1.3E-04** | 30 | 1.16 | 0.8 | 9.7E-01 |
|  |  |  |  |  |  |  |  |  |
| GO:0032502~developmental process | **503** | **21.90** | **1.6** | **1.9E-32** | 238 | 9.27 | 0.6 | 1.0E-00 |
| GO:0009888~tissue development | **137** | **5.96** | **1.9** | **2.9E-13** | 50 | 1.95 | 0.6 | 1.0E-00 |
| GO:0048513~organ development | **321** | **13.97** | **1.7** | **3.1E-23** | 146 | 5.68 | 0.6 | 1.0E-00 |
| GO:0007399~nervous system development | **210** | **9.14** | **2.1** | **2.8E-28** | 80 | 3.12 | 0.7 | 1.0E-00 |
| GO:0007420~brain development | **94** | **4.09** | **2.8** | **1.5E-21** | 28 | 1.09 | 0.7 | 9.9E-01 |
| GO:0048705~skeletal system morphogenesis | **44** | **1.92** | **2.9** | **5.3E-11** | 21 | 0.82 | 1.2 | 3.2E-01 |
| GO:0007517~muscle organ development | **38** | **1.65** | **1.9** | **1.9E-04** | 11 | 0.43 | 0.5 | 1.0E-00 |

B. Pathways in embryonic craniofacial tissues.

|  | Count | % of genes | Fold Enrichment | P value | Count | % of genes | Fold Enrichment | P value |
| --- | --- | --- | --- | --- | --- | --- | --- | --- |
|  | BEN target genes | | | | TFII-I target genes | | | |
|  |  |  |  |  |  |  |  |  |
| P00010:B cell activation | 12 | 0.52 | 1.3 | 3.4E-01 | 0 | 0.00 |  |  |
| P00053:T cell activation | 13 | 0.57 | 0.9 | 7.7E-01 | 7 | 0.27 | 0.7 | 9.5E-01 |
| P04393:Ras Pathway | 0 | 0.00 |  |  | 6 | 0.23 | 0.9 | 8.4E-01 |
| mmu04370:VEGF signaling pathway | 11 | 0.48 | 1.4 | 2.7E-01 | 5 | 0.19 | 0.4 | 1.0E-00 |
| NF-kB Signaling Pathway | 3 | 0.13 | 1.0 | 8.1E-01 | 0 | 0.00 |  |  |
| P00052:TGF-beta signaling pathway | **27** | **1.18** | **1.6** | **1.0E-02** | 5 | 0.19 | 0.4 | 1.0E-00 |
| P00048:PI3 kinase pathway | **20** | **0.87** | **1.6** | **4.1E-02** | 4 | 0.16 | 0.4 | 1.0E-00 |
