# Supplementary material for: Diversity and Complexity in Chromatin Recognition by TFII-I Transcription Factors in Pluripotent Embryonic Stem Cells and Embryonic Tissues
Source: PLoS One. 2012 Sep 10;7(9):e44443. doi: 10.1371/journal.pone.0044443 (PMC3438194; doi:10.1371/journal.pone.0044443)
Supplement: Table S12 — TFII-I transcription factors target a large set of developmental regulators. (DOC) [file pone.0044443.s016.doc]

Supplemental Table 12. The relative enrichment of developmental regulators among TFII-I and BEN target genes. The chi-squared test of the observed over- or under-represented genes is shown. p-value indicates a standard deviation from the expected value. ns – not significant. ESCs, mouse embryonic stem cells; ET, embryonic craniofacial tissues.

|  | **577 BEN target genes**  **in ESCs** | **5024 TFII-I target genes**  **in ESCs** | **1413 BEN target genes**  **in ET** | **970 TFII-I target genes**  **in ET** |
| --- | --- | --- | --- | --- |
|  |  |  |  |  |
|  | **Number of really found genes (number of expected genes)** | | | |
| **497 genes linked to the**  **craniofacial development** | **20 (17)**  **ns** | **197 (144)**  **p<0.05** | **162 (40)**  **p<0.0001** | **44 (28)**  **p<0.05** |
| **366 neural tube closure defect -**  **associated genes** | **15 (12)**  **ns** | **129 (106)**  **ns** | **123 (30)**  **p<0.0001** | **23 (20)**  **ns** |
| **739 genes associated with**  **bone/skeletal phenotype** | **33 (25)**  **ns** | **307 (214)**  **p<0.0001** | **217 (60)**  **p<0.0001** | **70 (41)**  **p<0.05** |
| **976 genes involved in**  **brain development** | **30 (32)**  **ns** | **368 (283)**  **P<0.0005** | **269 (79)**  **p<0.0001** | **70 (55)**  **ns** |
